# The impact of pre-evacuation ultrasound examination in histologically confirmed hydatidiform mole in missed abortion

**DOI:** 10.1186/s12905-020-01064-9

**Published:** 2020-09-10

**Authors:** Yunhui Tang, Chenqi Zhu, Chen Zhu, Feng Liang, Arier Lee, Xiaoying Yao, Qi Chen

**Affiliations:** 1 Department of Family Planning, The Hospital of Obstetrics & Gynaecology, Fudan University, 419 Fangxie Road, Shanghai, China; 2 Department of Gynaecology, Maternity and Child Health Care of ZaoZhuang, ZaoZhuang, Shandong China; 3 Section of Epidemiology and Biostatistics, School of Population Health, The University of Auckland, Auckland, New Zealand; 4 The Department of Obstetrics & Gynaecology, The University of Auckland, Auckland, New Zealand

**Keywords:** Hydatidiform mole, Ultrasound, β-hCG, Complete hydatidiform mole, Partial hydatidiform mole

## Abstract

**Background:**

Early detecting hydatidiform mole in missed abortion is challenge. In this retrospective observational study, we analysed the sensitivity of detecting hydatidiform mole by pre-evacuation ultrasound examination or naked eye after surgical uterine evacuation in missed abortion.

**Methods:**

Data on 577 cases with histologically confirmed hydatidiform mole were collected over a 10-year period and analysed. Data included serum β-hCG level before surgical evacuation, the ultrasound examination findings, histology findings and naked eye findings. In addition, serum β-hCG level on 2398 cases without hydatidiform mole was also collected.

**Results:**

The median maternal age was 29 (range, 17–53) years and the range of gestational age was 6 to 12 weeks. The sensitivity of detecting hydatidiform mole by ultrasound examination or by naked eye was 25% or 60% respectively. This sensitivity was not increased by the combination of ultrasound and naked eye. There was no difference in the sensitivity of detecting subtypes of hydatidiform mole. The higher β-hCG level was seen in cases with hydatidiform mole, compared to cases without hydatidiform mole**.** However, there was a lot of overlap in the distributions of β-hCG between the two groups**.**

**Conclusions:**

In this study, we found lower sensitivity of detecting hydatidiform mole by ultrasound in missed abortion. β-hCG level was higher in hydatidiform mole than in non- hydatidiform mole in missed abortion. Although higher sensitivity of detecting hydatidiform mole is seen by naked eye (60%), in order to minimise missed opportunity of detecting hydatidiform mole, our study suggests that routine histopathological examination is necessary in missed abortion.

## Background

Hydatidiform mole is one of the most common complications of gestational trophoblastic diseases (GTD), which affects 0.6–1.1 per 1000 pregnancies [1]. Asia including China has a higher incidence of this disease [2], and we have recently reported that the cases of hydatidiform mole were significantly increased in China in the last decade [3]. Although more than 80% of hydatidiform mole has spontaneous remission, 10 to 15% of cases may develop into invasive moles and 2–3% of cases may develop into choriocarcinoma. In addition, women with previous hydatidiform mole have an increased risk of developing a second hydatidiform mole in the next pregnancy [4]. Therefore, hydatidiform mole needs to be closely followed up in clinical practice.

To date, although routine histopathological examination in miscarriage is a gold standard for diagnosis of hydatidiform mole [5], there are no histological criteria in identifying the causes of miscarriage. In addition to the lower incidence of hydatidiform mole in miscarriage [6], this may be a limitation for routine histopathological examination in miscarriage in most centers. Recently with the increasing performance of ultrasound examination either routinely in the first trimester of pregnancy or for management of early pregnancy complications [7], hydatidiform mole may be able to be detected before developing into invasive moles or choriocarcinoma. Studies suggested that around 40 to 56% of hydatidiform mole were able to be detected on pre-evacuation ultrasound examination in miscarriages in the United Kingdom [8–11]. However, this detection rate may vary depending on the individual clinician’s experience and equipment among the countries [9, 12].

Missed abortion, one of the types of miscarriage occurs when a fetus does not form or dies but the placenta is still growing and continues to release hormones. The incidence of missed abortion is approximately 1% in all pregnancies and about 80% of cases occur in the first trimester. Most cases of missed abortion are associated with chromosomal abnormalities, and one of the possible causes of missed abortion in the first trimester is hydatidiform mole [13]. However, studies about the sensitivity of detecting hydatidiform mole by ultrasound examination in missed abortion are limited, in particular in developing countries such as China.

Therefore, in this retrospective observational study, we analysed the sensitivity of detecting hydatidiform mole on pre-evacuation ultrasound examination in the first trimester or by naked eye after surgical uterine evacuation in women with missed abortion. All the data were collected from the largest university teaching hospital in China.

## Methods

This study was approved by the ethic committee of The Hospital of Obstetrics & Gynaecology of Fudan University, China.

### Study setting

This was a retrospective cohort study of 10,561 women with missed abortion who attended our early pregnancy unit in The Hospital of Obstetrics and Gynaecology, Fudan University, China from January 2008 to December 2017. Our hospital is the largest Obstetrics & Gynaecology university teaching hospital in China and services a diverse urban and rural population in Shanghai, the largest city in China with a population of 25 million. Based on the current hospital guideline, it is routine practice to submit products from surgical uterine evacuation for histological examination to confirm the presence of hydatidiform mole in all missed abortion.

During the study period of 10 years, 1486 cases with hydatidiform mole in the first trimester were identified by histopathological examination in women with missed abortion. Due to data availability, data on serum levels of β-human chorionic gonadotropin (hCG) at the time of attending our unit, pre-evacuation ultrasound examination findings, histology findings and naked eye findings after surgical uterine evacuation from cases with histologically confirmed hydatidiform mole were only collected from 577 cases from the hospital medical electronic data base. Data were then retrospectively analysed. In addition, as control, data on levels of β-hCG on 2398 gestational age matched women with none hydatidiform molar missed abortion were also randomly collected from the hospital medical electronic data base.

Missed abortion is diagnosed with an empty gestational sac or an embryo/fetus without cardiac activity using Philip HD11 model ultrasound. The ultrasound examination was performed by a senior radiologist with more than 10 years of experiences. The diagnostic criteria used for the diagnosis of hydatidiform mole by ultrasound examination include the presence of a typical bunch of grapes (cluster of grapes or snow-storm) following the international guideline [14]. Naked eye findings are defined as placentae with grapelike vesicles observed with the naked eye in products from surgical uterine evacuation. Naked eye examination of uterine specimen was performed by a senior pathologist with more than 10 years of experiences.

The quantitative levels of β-hCG were measured 1–2 days before evacuation, using the Access Immunoassay Systems according to the guideline from the manufacture (Beckman Coulter (Shanghai, China).

### Statistical analysis

The sensitivity of detecting hydatidiform mole on pre-evacuation ultrasound examination in the first trimester or by naked eye after surgical uterine evacuation were analysed using SAS software version 9.4 (SAS Institute Inc., Cary, NC, USA). The statistical difference in the sensitivity of detecting hydatidiform mole on pre-evacuation ultrasound examination or naked eye between subtypes of hydatidiform mole was assessed by Chi-square test using Prison software version 7. The difference in the levels of β-hCG in cases with hydatidiform mole and without hydatidiform mole were assessed by Mann-Whitney U test. Two side *P* value of less than 0.05 was considered statistically significant.

## Results

During the study period, a total of 10,561 women with missed abortion in the first trimester were included. Of them, 1486 (14%) cases were identified with hydatidiform mole by histopathological examination. Demographic data on women with hydatidiform mole are summarised in Table 1. The median maternal age was 29 years, ranging from 17 to 53 years. Of the cases with hydatidiform mole, 1248 (84%) cases were diagnosed with complete hydatidiform mole.

**Table 1:** The clinical parameters in women with hydatidiform mole in missed abortion (*n* = 1486)

Maternal age (years, median/range)	29 (17–53)
Complete hydatidiform mole (number, %)	1248 (84%)
Partial hydatidiform mole (number, %)	238 (16%)

Data on serum levels of β-human chorionic gonadotropin (hCG) at the time of attending to our unit, pre-evacuation ultrasound examination, histology findings and naked eye findings after surgical uterine evacuation were only available on 577 cases with hydatidiform mole. We then analysed the sensitivity of detecting hydatidiform mole by pre-evacuation ultrasound examination or by naked eye in these 577 cases. The gestational age for these cases were from 5 to 15 weeks and 40% of cases were under 8 weeks. 149 (25.8%) of cases were suspected by pre-evacuation ultrasound examination. In addition, after uterine evacuation, the placentae with grapelike vesicles were observed by the naked eye in 348 (60.4%) cases amongst the 577 cases (Table 2). The sensitivity of detecting hydatidiform mole by either pre-evacuation ultrasound examination or naked eye was 66.03% (381/577 = 66.0, 95% CI 62.0 to 69.9%), while the sensitivity of detecting hydatidiform mole by the combination of pre-evacuation ultrasound examination and naked eye was only 20.3% (117/577 = 20.3, 95%CI 17.1 to 23.8%).

**Table 2:** The sensitivity of detecting hydatidiform mole by pre-evacuation ultrasound examination or naked eye in histologically confirmed hydatidiform mole (*n* = 577)

		Number (%)	95%CL	
			Lower CL	Upper CL
Ultrasound examination	**suspected**	**149 (25.8%)**	**22.3%**	**29.6%**
	not suspected	428 (74.2%)	70.5%	77.7%
Naked eye	**suspected**	**349 (60.5%)**	**56.4%**	**64.5%**
	not suspected	228 (39.5%)	35.5%	43.6%
The combination of ultrasound and naked eye	**suspected**	**117 (20.3%)**	**17.1%**	**23.8%**
	not suspected	460 (79.7%)	76.2%	82.9%
Ultrasound or naked eye	**suspected**	**381 (66.0%)**	**62.0%**	**69.9%**
	not suspected	196 (34%)	30.1%	37.9%
Total number, %		577 (100%)		

We then further analysed the sensitivity of detecting hydatidiform mole by pre-evacuation ultrasound or naked eye according to the subtypes of hydatidiform mole (complete hydatidiform mole and partial hydatidiform mole). There was no statistical difference in the sensitivity of detecting subtypes of hydatidiform mole by either pre-evacuation ultrasound or naked eye (*p* = 0.646 or *p* = 0.361 respectively, Table 3).

**Table 3:** The sensitivity of detecting subtypes of hydatidiform mole by pre-evacuation ultrasound examination or naked eye in histologically confirmed cases with hydatidiform mole (*n* = 577)

		Complete hydatidiform mole (*n* = 505)	Partial hydatidiform mole (*n* = 72)	*P*-value (chi-square test)
Ultrasound examination (n, %)	**Suspected**	**132 (26.1%)**	**17 (22.1%)**	*P* = 0.646
	Not suspected	373 (73.9%)	55 (77.9%)	
Naked eye (n, %)	**suspected**	**309 (61.2%)**	**40 (51.9%)**	*P* = 0.361
	Not suspected	196 (38.8%)	32 (48.1%)	

**Fig. 1:**
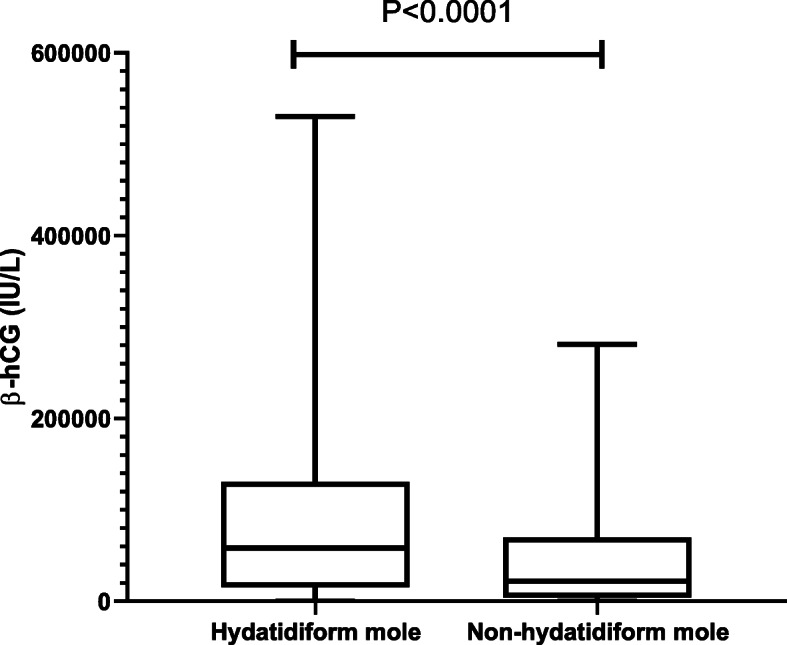
The comparison of the levels of β-hCG (IU/L) between cases with hydatidiform mole and without hydatidiform mole

To investigate whether the levels of β-hCG were different between cases with hydatidiform mole and cases without hydatidiform mole, we compared the levels of β-hCG between the two groups. As shown in Table 4, there were significantly higher levels of β-hCG in missed abortion with hydatidiform mole, compared with missed abortion without hydatidiform mole (Mann-Whitney U test *p*-value< 0.0001). However, there was a lot of overlap in the distributions of levels of β-hCG between the two groups (Fig. 1). We then compared the levels of β-hCG between cases with complete hydatidiform mole and cases with partial hydatidiform mole. There was no statistical difference in the median levels of β-hCG between cases with complete hydatidiform mole and with partial hydatidiform mole (63,211 vs 37,997 IU/L, *p* = 0.3756). We further compared the median levels of β-hCG between cases with complete hydatidiform mole and without hydatidiform mole. There was a significantly higher levels of β-hCG in cases with complete hydatidiform mole, compared to cases without hydatidiform mole (63,211 vs 21,988 IU/L, *p* < 0.0001).

**Table 4:** Levels of β-hCG (IU/L) in women with and without hydatidiform mole in missed abortion

	Mean	median	Min	mix
With hydatidiform mole (*n* = 506)^a^	78,919	58,194	1.9	530,486
Without hydatidiform mole (*n* = 2398)	45,691	21,988	3.28	281,134
*P* value^b^	< 0.0001			

^a^data on β-hCG in hydatidiform mole (*n* = 577) were not available in 71 cases

^b^Mann-Whitney U test

## Discussion

Hydatidiform mole is one of the causes in missed abortion and the majority of hydatidiform mole present as a miscarriage [9]. In this retrospective observational study with a relatively large sample size over a 10-year period, we demonstrate that the incidence of hydatidiform mole in missed abortion was 14% in our study population. The incidence of missed abortion among miscarriages is 2.6 to 9.4% [15, 16]. Other studies reported that the incidence of hydatidiform mole in missed abortion from 4 to 56% [8, 17]. Our data suggests that 0.4 to 1.4% of miscarriages could be due to hydatidiform mole.

With the increasing performance of ultrasound examination either routinely in the first trimester of pregnancy or for management of early pregnancy complications [18], a number of studies reported that approximately 40–50% of hydatidiform mole are detectable on pre-evacuation ultrasound examination in miscarriage [8–11], because the false-positive on diagnosis of hydatidiform mole by ultrasound examination is relatively low (less than 10%) [11]. However the accuracy of ultrasound examination for hydatidiform mole can be dependent on the expertise and experience in ultrasound examination of first trimester pregnancy complications between ultrasound operators or centers [9, 12]. In our current study, we found the sensitivity of detecting hydatidiform mole by ultrasound examination in missed abortion was significantly lower than in studies reported in the literature (25% vs 40–50%). The difference in the sensitivity of detecting hydatidiform mole between our current study and other studies [9, 11] could be because of the gestational age [7]. In our current study, there were around 40% of cases from early first trimester (under 8 weeks), while in other studies, the range of gestational age in the cases was 5 to 27 or 33 weeks. It is well-reported that detecting hydatidiform mole by ultrasound examination is difficult in early first trimester (before 8 weeks) [7]. In addition, ultrasound operator’s experience on ultrasound examination in China may be limited, compared with those in the United Kingdom.

Hydatidiform mole has two histological types including complete hydatidiform mole and partial hydatidiform mole. Studies reported that the ultrasound examination is less accurate on partial hydatidiform mole compared to complete hydatidiform mole [8, 9, 19], because the features of partial hydatidiform mole may not be present in the first trimester. However, in our current study we found that the sensitivity of diagnosis of two subtypes of hydatidiform mole by pre-evacuation ultrasound examination was not different. We do not know the exact reason for this difference between our study and others, but it is possible that some cases classified as partial mole could be early complete mole. It is also still could be due to the individual experience on ultrasound examination.

The naked eye examination at the time of uterine evacuation can indicate the first clue to detecting hydatidiform mole [19]. In our current study we found the sensitivity of detecting hydatidiform mole by naked eye after uterine evacuation was only 60% in all cases which were proven by histological examination later. Study reported that the combination of ultrasound and naked eye examination can reach to 80% for detecting complete hydatidiform mole and 30% for partial hydatidiform mole [19]. However, because of the lower sensitivity of detecting hydatidiform mole by ultrasound examination in our current study, the sensitivity of detecting hydatidiform mole by the combination of ultrasound examination and naked eye did not increased.

It is well-known that women with hydatidiform mole have higher levels of β-hCG and the levels of β-hCG may be a useful adjunct to histology in first trimester miscarriage [20]. Study reported higher β-hCG level in hydatidiform mole, compared to non- hydatidiform mole in missed abortion, suggesting the use of β-hCG as a screening tool for the diagnosis of hydatidiform mole in missed abortion [8]. In addition, in complete hydatidiform mole, the typical ultrasound features is associated with a higher levels of β-hCG [21]. In our current study, we also found significantly higher levels of β-hCG (IU/L) in cases with hydatidiform mole, compared with cases without hydatidiform mole in missed abortion. Although there was a lot of overlap in the distributions of the levels of β-hCG between cases with and without hydatidiform mole, we found higher levels of β-hCG in cases with complete hydatidiform mole, compared to cases without hydatidiform mole. Our finding may suggest that in missed abortion the β-hCG value was higher in hydatidiform mole than in non- hydatidiform mole, although there is an overlapping among them. Our finding also suggests that the levels of β-hCG prior to evacuation may be able to predict hydatidiform mole in missed abortion, in case when the specimen is not available for histological examination [22].

The routine histopathological examination in missed abortion is controversial. This is because to date, there are no histological criteria in identifying the causes of missed abortion. A recent study suggested that there is less value in performing histological examination in all miscarriages because of the lower incidence of hydatidiform mole and higher cost on histopathological examination [6]. However due to the lower detection rate of hydatidiform mole by ultrasound examination in missed abortion, other studies recommended that the routine histopathological examination for products from surgical uterine evacuation should be performed in order to identify and to follow up for future management of hydatidiform mole [5, 9, 17].

## Conclusion

In this retrospective study with a relatively large sample size, we found the sensitivity of detecting hydatidiform mole by pre-evacuation ultrasound examination is significantly lower (25%) than other studies reported in the literature (approximate 40–50%). Detecting hydatidiform mole by pre-evacuation ultrasound examination currently remains a diagnostic challenge, particularly for partial moles because the detection rate has not been improved in last decade [23]. Although the sensitivity of detecting hydatidiform mole by naked eye reached to 60%, in order to minimise missed opportunity of detecting hydatidiform mole, our study suggests that routinely performing histopathological examination is important in missed abortion, particularly in non-developed countries. However, the levels of β-hCG in missed abortion with hydatidiform mole, in particular in complete hydatidiform mole are significantly higher than cases without hydatidiform mole, suggesting β-hCG value prior to or after evacuation may be able to be used as a predictor of hydatidiform mole in missed abortion, particularly in cases without available specimen.

## Data Availability

The datasets used and/or analysed during the current study available from the corresponding author on reasonable request.

## Abbreviations

GTD: Gestational trophoblastic diseases
β- hCG: Beta human chorionic gonadotropin
